# Randomised Evaluations of Accepted Choices in Treatment (REACT) trials: large-scale pragmatic trials within databases of routinely collected electronic healthcare records

**DOI:** 10.1186/1745-6215-12-S1-A104

**Published:** 2011-12-13

**Authors:** Tjeerd-Pieter van Staa, Ben Goldacre, Martin Gulliford, Jackie Cassell, Munir Pirmohamed, Adel Taweel, Brendan Delaney, Liam Smeeth

**Affiliations:** 1 General Practice Research Database, Medicines and Healthcare products Regulatory Agency, London, UK; 2 Utrecht Institute for Pharmaceutical Sciences, Utrecht University, Utrecht, The Netherlands; 3 London School of Hygiene & Tropical Medicine, London, UK; 4 Primary Care and Public Health Sciences, King's College, London, UK; 5 Division of Primary Care and Public Health, Brighton and Sussex Medical School, University of Brighton, Brighton, UK; 6 The Wolfson Centre for Personalised Medicine, Institute of Translational Medicine, University of Liverpool, Liverpool, UK

## 

Pragmatic trials determine the effects of an intervention under the usual conditions in which it will be applied. Databases of electronic health records (EHR), such as the General Practice Research Database (GPRD), provide a unique opportunity to conduct large scale pragmatic trials. This paper describes the infrastructure as being implemented for two feasibility pragmatic trials within GPRD. Clinicians willing to participate first need to complete a web-based training (covering both the study protocol and key aspects of good clinical practice). The EHR database will be searched for potentially eligible patients and a list including codes of potentially eligible patients and those of clinicians who completed the training will be compiled. This list will then be sent, using secure file transfer, to the desktop computer of the clinician. When a potentially eligible patient then visits the clinician for a consultation, a flag at the clinician’s computer screen may then appear, notifying of the possibility to recruit. This flag provides a link to the study website, including the consent form and patient information sheet. After obtaining consent, the patient can then be randomised (using the website) and a prescription for the randomly allocated treatment can then be issued by the clinician. Daily downloads of the anonymous EHR records from the clinician’s office to the central research site will allow the reporting of suspected side-effects. The trial database can be compared periodically to the full GPRD for purposes of fraud detection and comparison of the trial patients to the ‘real-life population of users. GPRD is linked periodically to other NHS datasets (including hospital episode statistics, disease registries and death certificates), using an anonymous process through a trusted third party. This linkage allows an anonymous long-term data collection of major clinical outcomes with no intrusion. It is possible to contact the clinician in order to confirm the data as provided by the various sources. One trial concerns a comparison of two statins by randomising patients with primary hypercholesterolaemia and high CVD risk between simvastatin and atorvastatin (with 300 patients; ISRCTN33113202). The other trial concerns a comparison of antibiotic to no treatment in patients with a COPD exacerbation and non-purulent sputum (with 150 patients; ISRCTN72035428).

